# Hypothalamus-liver talks: whispers in the language of metabolism

**DOI:** 10.1007/s11154-026-10031-y

**Published:** 2026-03-30

**Authors:** Vitor Ferreira, Iara Fernández-González, Jane Jose Vattathara, Amanda Rodríguez-Díaz, Paola Fernández-Sanmartín, Carlos Diéguez

**Affiliations:** 1 Department of Physiology, CiMUS, University of Santiago de Compostela, Santiago de Compostela, 15782 Spain; 2 CIBER Fisiopatología de la Obesidad y Nutrición (CIBEROBN), Santiago de Compostela, 15706 Spain

**Keywords:** Hypothalamus, Liver disease, Obesity, Diabetes, Insulin, Amylin, Glucagon, GLP-1, FGF21

## Abstract

Closely associated with the exponential increase of obesity and sedentary life, liver-related disorders are a major global health concern. Recent data suggest that the global prevalence of metabolic dysfunction-associated steatotic liver disease (MASLD) among adults is of 32% and 5.1% for alcohol-related liver disease, with hepatic disorders contributing to 4% of global mortality, accounting for approximately 2 million deaths annually. Over the past two decades, the hypothalamus has emerged as a central hub in regulating whole body metabolic and energy homeostasis. Nevertheless, the interactome between the hypothalamus and the liver in the progression of liver metabolic dysfunctions, as well as its potential as a therapeutic target, remains poorly understood. In this review, we provide a comprehensive overview of the current knowledge regarding the hypothalamus-liver crosstalk, with a particular emphasis on the mechanisms underlying it. We explore how signals transmitted by different hormones can modulate these interactions, shedding light on their functional implications for hepatic regulation and systemic homeostasis through central signals.

## Hypothalamus–liver crosstalk: a quick dive

In the 1960 s, Shimazu and coworkers have shown for the first time that signals emerging from a small brain region - the hypothalamus - and transmitted through the autonomic nerves were able to regulate hepatic glycogen metabolism [1–3], demonstrating that central signals could control liver metabolism. Currently, a lot has been done to uncover the pathways that govern the interactome between the hypothalamus and the liver [4–6]: (a) Electrical stimulation and surgical denervation approaches, alongside pharmacological inhibition of the autonomic nerves, evidenced the relevance of these neuronal connections in driving hepatic metabolic functions [4]. (b) Additionally, immunohistochemistry and advanced electron microscopy have demonstrated that classical neurotransmitters (acetylcholine and noradrenaline), as well as neuropeptides (neuropeptide Y (NPY), glucagon-like peptide-1 (GLP-1), somatostatin, serotonin, among others) were found in the terminals of autonomic nerves in the liver, and are known to impact hepatic metabolism [4, 7, 8]. Lastly, (c) single-cell/single-nuclei transcriptomics is allowing to uncover the functional heterogeneity of specific hypothalamic neurons that are genetically, histologically and functionally different. In this review, we will focus on the insights of this bidirectional crosstalk between the hypothalamus and the liver, first by revising the neuronal pathways a mediate this communication, and then discussing the pancreatic and gastrointestinal hormonal mediators - insulin, glucagon, amylin, GLP-1and Fibroblast Growth Factor 21 (FGF21) - that play crucial role in conveying systemic nutritional status and energy demands.

## The hypothalamus-liver axis: harmonizing neurometabolic communication

**Fig. 1:**
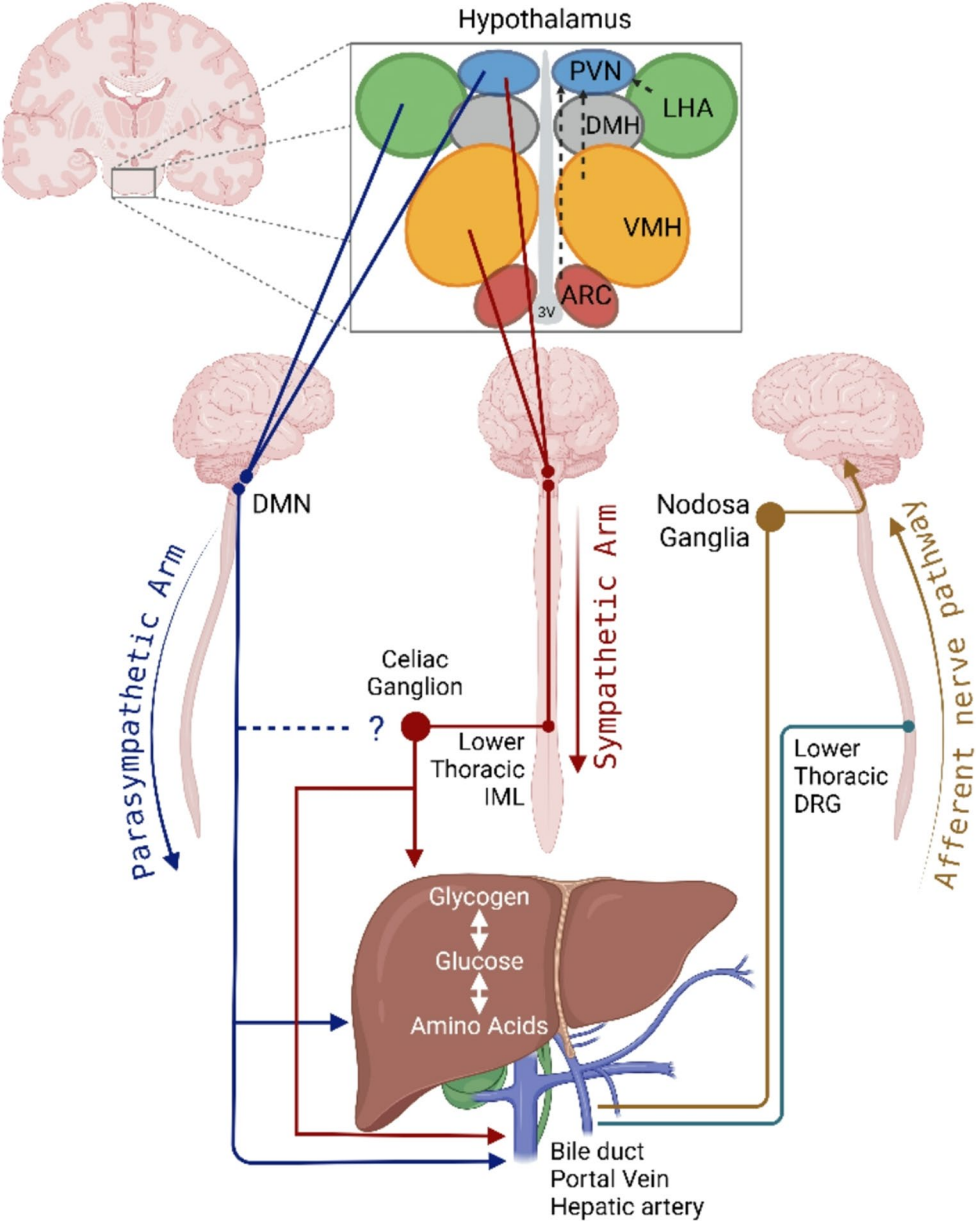
Efferent and afferent nerve pathways linking the hypothalamus and the liver. At the top, a schematic representation of the hypothalamus highlights its individual nuclei. At the bottom, from left to right, the parasympathetic branch (blue line) and the sympathetic branch (red line) of the efferent nerves pathways are depicted, transmitting signals from the hypothalamus to the liver. On the right, an outline of the afferent nerve pathways between the liver and hypothalamus: the brown line represents the vagal afferent pathway, while the turquoise line represents the spinal afferent nerve pathway. PVN: periventricular nucleus of the hypothalamus; LHA: lateral hypothalamic area; VMH: ventromedial nucleus; DMH: dorsomedial nucleus of the hypothalamus; ARC: arcuate nucleus of the hypothalamus; 3 V: third ventricle; DMN: dorsal motor nucleus of the hypothalamus; IML: intermediolateral column; DRG: dorsal root ganglia

The hypothalamus presents a particular cytoarchitecture, being organized in defined clusters of neurons called nuclei, that are interconnected via axonal projections, creating highly specialized neuronal networks (Fig. 1) [9–11]. Some hypothalamic nuclei are widely recognized for their roles in regulating metabolic processes. The arcuate nucleus (ARC), the ventromedial hypothalamus (VMH), the lateral hypothalamic area (LHA), and the paraventricular nucleus of the hypothalamus (PVN) all contribute to the control of feeding behavior, energy expenditure, and glucose homeostasis. Together, these nuclei form a complex and dynamic network essential for maintaining metabolic balance. Related to their ability to impact hepatic function, the most investigated hypothalamic nuclei are the LHA, the VMH and the PVN [12–14].

The hypothalamus and liver play essential roles for homeostasis preservation, engaging in continuous bidirectional communication through neural pathways, hormonal signals, and other molecular mediators [4, 12, 15]. In the process of maintaining metabolic homeostasis, the system adapts to changes in nutritional status and energy requirements, being this regulation mainly carried out by the autonomic nervous system signals and pituitary hormones [12, 16]. Of interest, the efferent nerves can be part of the sympathetic and parasympathetic systems and their pathways emerge from different hypothalamic areas: VMH [17, 18], LHA [17, 18], PVN [6] and the ARC [19] (Fig. 1).

The VMH is by excellence the region that governs sympathetic outflow [20, 21] and interacts intricately with the liver, despite lacking direct synaptic connections with hepatic vagal motor neurons. Instead, steroidogenic factor 1 (SF1)-positive neurons (whose expression is highly restricted to the VMH [22]) project to key autonomic centers in the brainstem. Particularly, the major VMH projections to the liver go through the medullary reticular formation, that integrate hypothalamic signals and relay them to sympathetic preganglionic neurons in the thoracolumbar spinal cord. From there, sympathetic efferents synapse in prevertebral (collateral) ganglia within the abdominal cavity, before postganglionic fibers enter the liver along the hepatic vasculature (Fig. 1) [17, 18]. The activation of this pathway promotes hepatic glucose output by glycogenolysis, and enhances phosphoenolpyruvate carboxykinase (PEPCK) activity, a key enzyme in gluconeogenesis, while it suppresses the glycolytic enzyme pyruvate kinase (PK), resulting in depletion of hepatic glycogen content and hyperglycemia [17, 23–25]. Additionally, Liu et al. described that corticotropin-releasing hormone (CRH) receptor stimulation suppresses VMH neuronal inhibitory tone by disrupting gamma-aminobutyric acid (GABA) receptors membrane trafficking, promoting VMH-sympathetic nerve-liver signals that mediate CRH-induced glucose release [26–28].

As for the PVN, it is a heterogenous collection of neurons with recognized neuroendocrine, autonomic and behavioral actions [29, 30]. The PVN acts to coordinate and consolidate inputs from the other hypothalamic nuclei, including the suprachiasmatic nucleus (SCN) and the ARC, serving as the principal hypothalamic motor output system [30]. In this line, the PVN integrates information from other hypothalamic nuclei involved in the modulation of hepatic functions [26] and sends signals both via intermediolateral nucleus in the spinal cord (sympathetic nervous system), and dorsal nucleus vagus in the brainstem (parasympathetic nervous system) [6, 31]. Therefore, the PVN seems to be involved in both parasympathetic and sympathetic regulation of hepatic metabolism (Fig. 1). Of interest, the stimulation of estrogen receptor alpha (ERα)-expressing glutamatergic neurons in the VMH projecting to PVN, activates PVN sympathetic pre-autonomic neurons, and enhances sympathetic tone to the liver [32]. In contrast to the VMH, which regulates the hepatic function indirectly through interactions with other hypothalamic nuclei and downstream brain regions, the action of PVN seems to be more direct through the autonomous nervous system.

Regarding the connections with the LHA, these are directly aimed to the parasympathetic cell groups of the nucleus vagus/ambiguous complex, which supply the parasympathetic nerves to the liver constituting the hepatic branch of the vagal nerves [17, 18], and also to the nearby medullary reticular cell group (Fig. 1). LHA stimulation decreases appetite and enhances anabolic responses, including enhanced insulin secretion, which suppresses PEPCK activity in the liver [25] and activates hepatic glycogen synthase [17, 23, 24, 33], thereby reducing gluconeogenesis. Within this framework, the roles of VMH and LHA exhibit, simultaneously, both opposing and complementary characteristics [4, 34].

Liver sensory neurons are responsible for sending feedback to the hypothalamus, mainly to the ARC and VMH, on the metabolic state of the liver and, as a proxy, of the whole organism (e.g., glucose, lipid and amino acid levels) [19, 26]. The hypothalamus integrates this peripheral information, monitoring liver function and promoting an appropriate response [19, 26]. Interestingly, the afferent innervation of the portal hepatic area has not been studied as extensively as that of other major organs. Generally, it is accepted that, unlike noradrenergic sympathetic efferent nerves, sensory nerve fibers do not directly innervate hepatocytes but are instead restricted to the stroma surrounding the hepatic vascular and biliary triads, as well as to extrahepatic portions of the portal vein and bile ducts [35]. This anatomical arrangement might explain why internal hepatic damage produces no pain, discomfort arises only when the Glisson’s capsule is distended, activating pain receptors in that membrane [36, 37]. Afferent nerves are categorized into two distinct pathways. The spinal afferent pathway conveys hepatic nociceptive information to the brain and underwrites pathological pain states (mainly visceral), while also participating in mechanisms of homeostatic control, such as water and nutrient balance [4, 13, 26, 35, 38]. The vagal afferent nerve pathway [4], in turn, detects circulating metabolites, including glucose [39], lipids [40], amino-acid [41], and cytokines [42]. These nerves have been shown to be particularly important for the circadian regulation of feeding, as disturbances in the liver clock alter hepatic vagal afferent signaling and disrupt feeding behavior in mice [43]. High-fat diet (HFD) feeding produces similar alterations in liver rhythms and feeding control, whereas preventing hepatic feedback by surgically severing the vagus nerve limits HFD-induced weight gain [43]. The importance of vagal sensory neurons was further demonstrated by Hwang and colleagues, who showed that loss of this liver-brain axis prevented diet-induce obesity, increased energy expenditure and improved glucose homeostasis in mice [44]. Together, these studies highlight hepatic afferent nerves as essential for the regulation of feeding behavior and lipid and glucose homeostasis [45]. Additionally, beyond the brain-liver direct neural connections, the autonomic nerves can centrally modulate hepatic metabolism indirectly, through interactions with endocrine organs, like the adrenal glands and pancreas [4].

Given their essential role in mediating communication between the periphery and the central nervous system (CNS), this review will focus on the intricate mechanisms through which selected pancreatic and gastrointestinal hormones regulate hepatic metabolism through central actions. We will examine the specific pathways underlying these effects and discuss their boarder implications for maintaining systemic homeostasis, with a particular emphasis on hepatic metabolic dysfunctions [46]. This topic is especially relevant in light of the exponential risen incidence of obesity and metabolic dysfunction-associated steatotic liver disease (MASLD). MASLD is characterized by excessive hepatic lipid accumulation (hepatic steatosis), which can lead to inflammation (steatohepatitis) and progressive fibrosis [47]. It refines and expands the concept of non-alcoholic fatty liver disease (NAFLD) by incorporating cardiometabolic risk factors. Moreover, the severity and progression of MASLD are influenced by multiple factors, including genetic susceptibility, adiposity, dietary composition, insulin resistance, gut microbiome, and a wide range of endocrine effectors [47]. Within this frame, the hypothalamus (and the disturbances in its neurocircuitries in response to pathological conditions, such as obesity and T2D) plays a relevant role in the development and progression of hepatosteatosis, insulin resistance and MASLD [48–52]. Beyond its well-stablished role in regulating hunger/satiety, food preference and adipose tissue thermogenesis/browning [10, 11, 53–55], the hypothalamus also directly influences peripheral lipid metabolism and fluxes. For instance, activation of the melanin-concentrating hormone (MCH) receptors in LHA promotes hepatic steatosis through parasympathetic nervous system signaling, while MCH receptors activation in ARC increases fat deposition in white adipose tissue via suppression of sympathetic outflow [52].

It is important to highlight that, besides the pancreatic and gastrointestinal hormones discussed throughout this review, other hormones known to play significant roles in the central regulation of hepatic metabolism - such as thyroid hormones, estrogens or ghrelin - are reviewed in separate chapters of this issue [56–59].

## Hypothalamic echoes: hormonal signals modulating hepatic function

### Hormones synthetized by the pancreas

#### Insulin

**Fig. 2:**
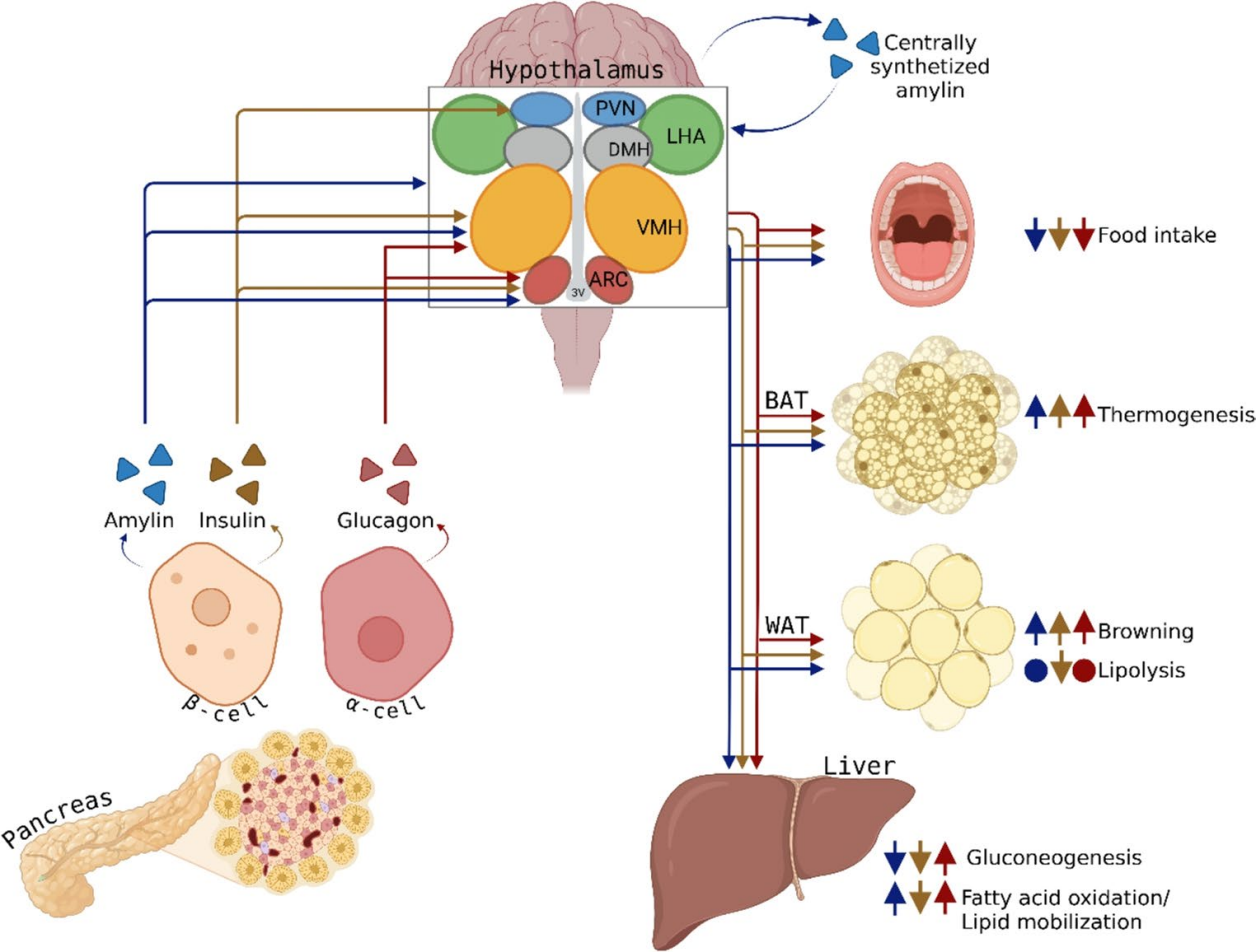
Central-peripheral interactome driven by pancreatic hormone-hypothalamic signaling. At the top of the diagram, a schematic representation of the hypothalamic nuclei is shown. Insulin and amylin are produced by pancreatic β-cells, while glucagon is synthetized in α-cells. In addition to their direct effects on peripheral tissues, these hormones act on the different hypothalamic nuclei, modulating feeding behavior, adipose tissue function, and hepatic lipid and glucose metabolism. PVN: periventricular nucleus of the hypothalamus; LHA: lateral hypothalamic area; VMH: ventromedial nucleus; DMH: dorsomedial nucleus of the hypothalamus; ARC: arcuate nucleus of the hypothalamus; 3 V: third ventricle; BAT: brown adipose tissue; WAT: white adipose tissue

Insulin is a 51 amino-acid peptide hormone synthetized and secreted by pancreatic β-cells (Fig. 2) [60, 61] in response to nutrient availability, particularly glucose [62]. It plays a central role in metabolic homeostasis. Importantly, the regulation of hepatic metabolism by insulin is critical for glucose homeostasis, as the liver is the primary source of glucose production during fasting [63]. While the peripheral actions of insulin are well established, this review will focus on its centrally mediated mechanisms that influence liver function in relation to energy and metabolic homeostasis.

For a long time, insulin and insulin receptor (IR) signaling were thought to be restricted to peripheral tissues, and the brain was traditionally considered to be an insulin-insensitive organ. This assumption stemmed largely from the poor correlation between circulating insulin levels and whole-brain glucose uptake [64]. In 1978, however, Havrankova and colleagues challenged this view by discovering insulin and its receptor in the brain [65]. It is now well stablished that insulin crosses the blood-brain barrier (BBB) through a saturable, carrier-mediated transcytosis system involving endocytosis and potentially other transporters beyond the classical IR. Recent data indicates that tanycytes largely mediate insulin entry into the hypothalamus, thereby influencing insulin interaction with hypothalamic neurons and, consequently, energy and metabolic balance [66]. Once in the brain, insulin acts through its specific receptor. Of interest, IR is a heterotetrametric protein with intrinsic tyrosine kinase, composed of four chains: two extracellular α-subunits and two intracellular β-subunits linked by disulfide bonds [67]. Binding of insulin to the α-subunits induces a dose-dependent autophosphorylation of three tyrosine residues within the activation loop of the kinase domain in the β-subunits, fully activating the tyrosine kinase function of the receptor [64, 68]. Two structurally and functionally distinct IRs isoforms exist: IR-B which predominates in adult peripheral tissues such as muscle, liver, kidney and adipose tissue, and a shorter isoform IR-A, generated by alternative splicing of exon 11 that removes 12 amino acids from the C-terminus of the α-subunit [64, 69]. Consistent with their roles in the regulation of energy and metabolic homeostasis, autonomic control of liver and adipose tissue, as well as memory and cognitive function, both isoforms are expressed within the brain, with particularly high expression in the hypothalamus and the hippocampus [64, 70–74]. Intracerebroventricular (ICV) or region-specific insulin administration in rodents reduces body weight by decreasing food intake and increasing energy expenditure (Fig. 2), effects abolished in neuronal IR knockout models or under central insulin resistance. These findings are physiologically significant, as passive immunization against central insulin or genetic silencing of IRs induces hyperphagia and obesity [75, 76]. Consistent with this, neuron-specific IR knockout mice exhibit impaired thermogenic response to cold exposure [77, 78]. In humans, intranasal insulin administration enhances the acute thermoregulatory and glucoregulatory response to food intake [79].

The role of central insulin in whole body glucose homeostasis is beyond any doubt. Functional magnetic resonance revealed that men with high hypothalamic insulin sensitivity display enhanced second-phase insulin secretion from pancreatic β cells [80]. Beyond indirect hypothalamic regulation of pancreatic insulin secretion, central signals may also directly influence hepatic metabolism [81]. Indeed, central insulin suppresses hepatic glucose production by modulating gluconeogenesis and glycogenolysis gene expression and preventing lipid accumulation, independently of food intake [82, 83]. Since insulin has been shown to promote hyperpolarization of both ARC neuronal populations, agouti-related peptide (AgRP) and proopiomelanocortin (POMC) neurons, it was hypothesized that these neurons are crucial targets of insulin signaling [84]. In support of this idea, deletion of the IR in AgRP neurons, but not in POMC neurons, impairs insulin’s ability to suppress hepatic glucose production without altering whole body adiposity or insulin-mediated suppression of lipolysis in adipose tissue [85–88]. Moreover, deletion of the IR in either AgRP or POMC neurons worsens glucose tolerance under HFD conditions. Nevertheless, HFD-fed mice lacking the IR specifically in POMC neurons, but not those with AgRP-specific IR deletion, exhibited increased hepatic triglyceride accumulation [86].Together, these findings suggest that while AgRP neurons are essential for maintenance of glucose homeostasis, insulin signaling in POMC appears to influence lipolytic regulation and contributes to HFD-induced hepatosteatosis. A step further, Martins dos Santos and coworkers demonstrated through transsynaptic retrograde tracing that PVN neurons are connected to the liver [89]. This evidence, associated with the fact that insulin stimulates the firing frequency of PVN neurons, an effect inhibited by rapamycin pretreatment [89], supports the hypothesis that PVN neurons may constitute part of the parasympathetic network innervating the liver and regulating hepatic functions. Moreover, selective deletion of IR in SF1 neurons of the VMH protected mice against HFD-induced obesity and insulin resistance, while improving leptin sensitivity [90]. Given that the majority of insulin-responsive VMH neurons are in the same area that provides glutamatergic innervations to anorexigenic POMC neurons in the ARC, it is plausible that the hyperactivation of insulin signaling in the VMH under HFD conditions, may inhibit these projections [90].

In summary, central insulin action is pivotal in modulating behaviors and systemic metabolism [91] (Table 1). The presence of the IR in multiple brain regions, particularly the hypothalamus and the midbrain in both mice and human [91], illustrates the importance of central insulin signal in regulating feeding, thermogenesis, gluconeogenesis and lipolysis, and by it, whole-body metabolism. Notably, central insulin resistance induced by overnutrition precedes peripheral insulin dysfunction, suggesting that impaired brain insulin response may play an early and crucial role in the molecular mechanisms that lead to dyslipidemia, hyperglycemia and metabolic syndrome.

However, despite neuroimaging evidence linking central insulin sensitivity to eating behavior and body weight regulation through modulation of hypothalamic and reward-related blood oxygen level-dependent (BOLD) responses to food cues [92], human studies using intranasal administered insulin (as a brain-specific insulin delivery method) combined with pancreatic clamps have demonstrated weaker inhibitory effects on hepatic gluconeogenesis compared with the preclinical findings [93–96]. Thus, although a targeted approach to enhance central insulin action may represent a promising therapeutic strategy to address lipid dysregulation and lipotoxicity in metabolic disorders, its efficacy remains uncertain [96]. In this regard, intranasal insulin administration may be ineffective in the cases where hypothalamic insulin resistance stems from receptor downregulation or impaired signaling due inflammation, lipotoxicity or genetic alterations [96]. Collectively, current evidence highlights that central insulin effects - mediated through vagal and sympathetic pathways - can occur independently of peripheral insulin levels or direct hepatic IR activation, underscoring a crucial CNS-to-liver regulatory axis.

#### Glucagon

Glucagon, a 29 amino-acid peptide hormone synthetized and secreted by pancreatic α-cells (Fig. 2), exerts paradoxical yet complementary effects with insulin in maintaining blood glucose levels by promoting hepatic glucose output through gluconeogenesis and glycogenolysis. Beyond its well-known role in type 1 and T2D, dysregulation of glucagon pathways has been implicated in the pathogenesis of metabolic dysfunction-associated steatotic liver disease (MASLD) and chronic kidney disease (CKD) – conditions frequently linked to hyperglucagonemia and hepatic glucagon resistance [97–101]. These findings have led to the proposal of the “glucagonocentric hypothesis”, which repositions glucagon as more than a counter-regulatory hormone to insulin, highlighting its potential as a co-drive of disease.

As in the periphery, the interplay between glucagon and insulin signaling in the hypothalamus is essential for systemic glucose homeostasis and lipid metabolism [102–104]. These central mechanisms are closely connected to hepatic signaling pathways, underscoring the integrative role of brain-liver communication in metabolic regulation. Glucagon receptors are expressed in the brain at much lower levels than in traditional metabolic tissues such as the liver, kidney, and adipose tissue. Within the CNS, glucagon receptor expression is most prominent in hypothalamic nuclei like the ARC and PVN, with lower levels in the VMH and brainstem. Recently gleaned data supports a relevant role for central glucagon in modulating energy balance, glucose homeostasis and sympathetic outflow [105, 106]. Mechanistically, glucagon influences hypothalamic neuronal activity [107] by stimulating the adenylate cyclase, increasing cAMP levels [106] and, in turn, activating protein kinase A (PKA) [108].

It is well established that glucagon influences energy balance primarily by reducing meal size via a liver–brain vagal pathway and direct hypothalamic action [109] (Table 1). Passive immunization against glucagon in mice increases meal size, supporting its physiological role through vagal signals from the liver to the area postrema (AP)/nucleus of the solitary tract (NTS) and onwards to the hypothalamus [109, 110]. Central glucagon strongly suppresses feeding via ARC glucagon receptor and PKA– Ca2+/calmodulin-dependent protein kinase kinase β (CaMKKβ)– AMP-activated protein kinase (AMPK)–AgRP signaling, with effects observed at doses far lower than those required peripherally. In obesity, impaired CaMKKβ signaling contributes to resistance to the anorexigenic effects of glucagon [109]. Beyond reducing food intake, glucagon enhances energy expenditure by increasing brown adipose tissue (BAT) thermogenesis, likely mediated by fibroblast growth factor 21 (FGF21) and, at least in part, through CNS-sympathetic pathways [109, 111–113]. Notably, glucagon increases energy expenditure even in the absence of UCP1, thereby further contributing to body weight reduction.

While direct activation of glucagon receptors in hepatocytes decreases de novo lipogenesis, increases lipolysis, and enhances fatty-acid oxidation, thereby improving steatosis in obese MASLD models, the role of glucagon signaling in the CNS in regulating whole-body lipid metabolism remains less clear. The direct anorexigenic effect of glucagon decreases food intake, which secondarily lowers hepatic lipid influx and improves steatosis in diet-induced obesity. Although studies using central glucagon-like peptide 1 (GLP-1)/glucagon co-agonism have implicated hypothalamic nuclei and brainstem, direct evidence that selective central glucagon receptors activation alters hepatic de novo lipogenesis, fatty acid oxidation, or very low-density lipoprotein (VLDL) secretion independently of food intake is still lacking [97, 109].

Early studies revealed that central administration of a relatively high dose of glucagon (10 ng) in dogs elicited a biphasic effect on the circulating glucose levels, characterized by a transient hypoglycemia followed by hyperglycemia [114]. Intriguingly, hypoglycemia was absent in vagotomized dogs implicating a brain-liver axis in mediating the central glucagon-induced reduction in glucose levels, while pancreatectomy circumvented hyperglycemic response, highlighting pancreatic involvement [114]. Despite their importance, these early studies did not identify the specific hypothalamic nuclei responsible for glucagon-mediated regulation of glucose metabolism. More recent work has demonstrated that during fasting-induced hypoglycemia, glucagon action in AgRP neurons of the mediobasal hypothalamus (MBH) inhibits hepatic glucose production via glucagon receptors, PKA and K_ATP_ channels (Fig. 2) [105, 115]. Additionally, vagotomy abrogated these effects, confirming the requirement of intact vagal signaling. Furthermore, consistent with the classical role of glucagon in suppressing food intake (mediated via the liver-vagus nerve-hypothalamus axis) and enhancing energy expenditure (through BAT thermogenesis [97, 109]), hypothalamic glucagon resistance has been linked to hyperglycemia in diabetes and obesity [116].

Finally, the first dual agonist developed for the treatment of obesity was a chimera binding to both GLP-1 receptor (GLP-1R) and the receptor of glucagon. Chronic administration of this dual agonist produced striking reductions in adiposity and body weight in diet-induced obese mice, driven by decreased food intake and increased energy expenditure [117]. This seminal discovery has since inspired the development of an expanding portfolio of novel agents that are now progressing toward clinical use application.

#### Amylin

Amylin is a 37 amino-acid peptide co-secreted with insulin by pancreatic β-cells (Fig. 2), where it primarily functions to suppress glucagon secretion. Beyond its peripheral role, amylin is also synthesized in several CNS regions, including the amygdala, AP, nucleus accumbens (NAc), ARC, VMH and a subset of prodynorphin (Pdyn) neurons in the LHA, with expression showing sexual dimorphism under both chow or HFD [118]. Unlike GLP-1, amylin lacks incretin activity. Its principal physiological functions are mediated through specific heterodimeric receptors composed of the calcitonin receptor and one of three accessory proteins called receptor-activity modifying proteins (RAMP1, RAMP2 and RAMP3), generating three distinct amylin receptors subtypes: AMY1R, AMY2R, and AMY3R. These receptors are widely distributed across hypothalamic and brainstem regions, where their activation contributes to energy and metabolic homeostasis. Amylin acts as an anorexigenic signal, slows gastric emptying, and increases sympathetic tone to BAT, collectively promoting weight loss [119, 120]. In humans, coadministration of the GLP-1 agonist semaglutide with the amylin agonist cagrilintide produced ~ 24% body-weight reduction in overweight or obese adults, comparable to the most potent current therapies [121].

Additionally, amylin contributes to metabolic homeostasis through diverse mechanisms. Early studies identified its ability to stimulate glycogenolysis and gluconeogenesis in hepatocytes in vitro (Fig. 2) [122], although the physiological relevance of these peripheral effects remains controversial. More recent evidence emphasizes central actions of amylin, demonstrating that by acting at the CNS level, amylin plays a key role also in metabolic homeostasis, often independently of its effects on energy balance. Fundamentally, circulating glucose levels are primarily regulated by intestinal absorption during the postprandial state, glycogenolysis and gluconeogenesis. Centrally, amylin has been shown to slow gastric emptying, thereby reducing postprandial glucose peaks, and suppresses glucagon secretion via AP receptors and vagal efferent pathways, decreasing glycogenolysis and gluconeogenesis [123, 124]. Importantly, these effects are glucose-dependent, with hypoglycemia preventing the action of amylin on gastric emptying and glucagon secretion. Given these effects on energy and metabolic homeostasis, amylin’s involvement in liver disease appears evident. Indeed, preclinical studies demonstrate that amylin analogs reduce body weight, decrease hepatic fat deposition, and improve glucose tolerance and liver histology in diet-induced metabolic liver disease [125, 126]. Although amylin and its analogs – particularly in combination with GLP-1R-agonists - show promising potential for T2D and metabolic liver diseases such as MASLD [127, 128], large-scale clinical data specifically targeting liver disease remain limited.

In summary, amylin has emerged as a key component of the gut-brain axis, regulating energy balance and glucose homeostasis (Table 1). Its synthesis in multiple CNS regions, together with the distribution of amylin receptors, underscores the importance of its central actions. Notably, novel amylin analogs, including dual GLP-1/amylin receptors agonists, are currently being tested in humans for the treatment of both T2D and obesity, with encouraging results reported from recent phase III trials. Nevertheless, despite the growing evidence, our understanding on the regulation of amylin-expressing neurons and the central mechanisms mediating its effects remains scanty and requires further investigation.

### Gastrointestinal signals: *Glucagon-like peptide-1 (GLP-1)*

**Fig. 3:**
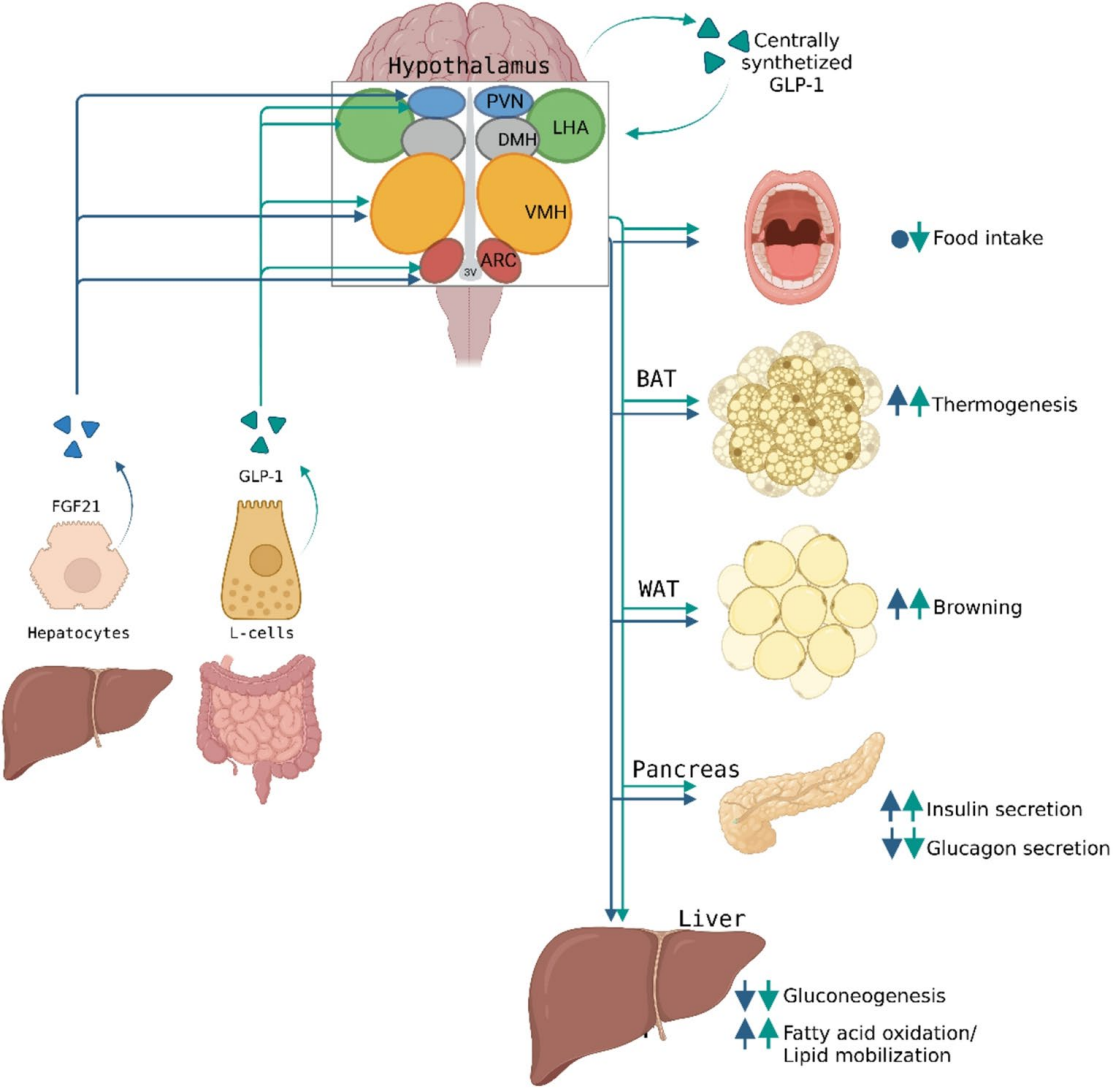
Central-peripheral crosstalk mediated by hypothalamic action of FGF21 and GLP-1. At the top of the diagram, a simplistic representation of the hypothalamic nuclei is shown. FGF21 is predominantly produced by hepatocytes, GLP-1 by the gut and the CNS. Beyond their direct effects on peripheral tissues, these hormones act on hypothalamic nuclei, modulating feeding, adipose tissue and pancreatic function, as well as hepatic lipid and glucose metabolism. PVN: periventricular nucleus of the hypothalamus; LHA: lateral hypothalamic area; VMH: ventromedial nucleus; DMH: dorsomedial nucleus of the hypothalamus; ARC: arcuate nucleus of the hypothalamus; 3 V: third ventricle; BAT: brown adipose tissue; WAT: white adipose tissue

GLP-1 is an incretin hormone with profound effects on systemic metabolism. It enhances β-cells proliferation, stimulates anti-apoptotic pathways and glucose-dependent insulin secretion, suppresses glucagon release, delays gastric emptying, and suppresses food intake (Fig. 3) [129–131]. The ability of GLP-1 to modulate pancreatic insulin and glucagon secretion is thought to underlie its capacity to reduce hepatic glucose output [132]. Since the liver does not present GLP-1R and direct GLP-1 and GIP exposure fail to impact hepatocytes and stellate cells in vitro [133], hepatic effects of GLP-1 are likely indirectly, probably mediated via the autonomic nervous system.

In this regard, GLP-1R is G-protein coupled receptor expressed in pancreatic β-cells, but also in neurons of multiple CNS regions [102], as well as in the kidney, lung, heart, adipose tissue, and other organs [129, 134, 135]. Within the CNS, GLP-1R is most abundant in areas involved in the regulation of energy balance and metabolism (hypothalamus and brainstem), reward circuits (VTA, NAc), autonomic output (e.g., VMH and PVN), and neuroendocrine function. Notably, GLP-1 is also synthesized by brainstem neurons, particularly in the NTS [136], and release into the hypothalamus, supporting potential central actions that modulate its peripheral effects. This was further supported by the increase c-Fos expression GLP-1-producing brainstem neurons following gastric distension [137], while vagal afferent denervation abolished GLP-1 effects on gastric emptying in rats [138]. The role GLP-1 in satiety control is undisputable, with hypothalamic signaling playing a key role in this context. Both ICV and peripheral administration of GLP-1R agonists promoted hypophagia and weight loss in preclinical models (Fig. 3) [139–141]. Recent evidence shows that distinct hindbrain GLP-1R circuits differentially regulate satiety and aversion. GLP-1R signaling from the NTS to the PVN suppresses food intake independently of nausea-like response, whereas GLP-1R activation in the AP projecting to the lateral parabrachial nucleus primarily drives aversive effects. Notably, disruption of aversion-related pathways does not eliminate the anorexigenic actions of GLP-1R agonists, highlighting NTS-derived GLP-1 signaling as a key contributor to their therapeutic efficacy [142]. Notably, the activation of GLP-1 neurons in the brainstem reduces basal hepatic glucose production and improves intraperitoneal glucose tolerance and hepatic insulin response [143]. In this line, recent studies have demonstrated that stimulation of NTS GLP-1 neurons suppresses glucose production without affecting its uptake [143, 144]. Conversely, the GLP-1R antagonist exendin-(9–39) induces weight gain [141], while reduced LHA GLP-1R expression leads to hyperphagia, weight gain and mild impaired glucose tolerance [104, 145]. Collectively, these findings highlight the central GLP-1 system as a regulator of both energy balance and glucose metabolism, with implications for hepatic health.

Beyond its effects on glucose homeostasis, central GLP-1 action also plays a key role in lipid metabolism [146]. ICV GLP-1 administration decreases triglyceride content in white adipose tissue (WAT) and liver, likely via reduced lipogenesis. While hepatic effects appear secondary to the anorectic effect of GLP-1, WAT effects seem to be mediated by sympathetic pathways independent of food intake, pointing that different central circuits drive the effects on these tissues. Interestingly, although in HFD-fed mice the anorectic responses to GLP-1 remain intact, the CNS GLP-1 system loses the ability to modulate adipocyte metabolism, indicating obesity-induced adipocyte resistance to CNS GLP-1 [146]. Whether this resistance can be overcome by targeting specific brain regions and/or therapeutic doses of GLP-1R agonists remains unclear. Notably, studies with liraglutide in mice show stimulation of BAT thermogenesis and WAT browning independent of nutrient intake, mediated by VMH signaling (Fig. 3). Importantly, VMH AMPK activation blunts these effects, while the reduction in body weight caused by the central injection of liraglutide in other hypothalamic sites is sufficiently explained by food intake suppression [147]. Taken together, this data suggest that GLP-1 regulates whole body lipid metabolism through several interplaying mechanisms, which include altered food intake, direct sympathetic BAT activation and WAT browning, and lipid mobilization as a response to a crosstalk between the CNS, WAT, BAT and the liver (Table 1).

Currently, given the striking physiological effects of GLP-1, GLP-1R agonists have gained major attention as potential pharmacological therapies for T2D and obesity [148]. Centrally, GLP-1 is largely produced in the brainstem and subsequently transported to key hypothalamic regions, decreasing food intake and body weight. In addition to suppress homeostatic food intake, GLP-1R agonists also suppress hedonic (reward-driven) feeding by acting on mesolimbic reward circuits, including the VTA, NAc and supramammilary nucleus-LHA pathways [149]. Furthermore, studies in experimental models show that GLP-1R agonists inhibit dopamine neuron activity in response to palatable food, effectively reducing hedonic feeding [150], while clinical data indicate improved control over eating, reduced emotional and cue-driven food intake, and decreased binge-eating symptoms [151]. GLP-1R activation enhances hyperglycemia-induced insulin secretion, delays gastric emptying, and is strongly recommended in the treatment of T2D [152] and obesity [153, 154]. In addition to improving glycemic control and body weight, GLP-1R agonists enhance insulin sensitivity, attenuate inflammation and reduce hepatic steatosis [155, 156]. These prominent effects arise the therapeutical potential of these compounds for MASLD [48, 157–160]. By combining central appetite regulation with direct hepatoprotective effects, GLP-1R agonists represent a dual-action strategy. In fact, Armstrong and coworkers have described in a phase 2 trial, that liraglutide improves steatosis and hepatocyte ballooning, with histological resolution of steatohepatitis in 39% of patients [161, 162]. Longer studies with semaglutide showed a dose-dependent resolution of non-alcoholic steatohepatitis (NASH) features without progression to fibrosis in 59% of the patients after 72 weeks [158]. Although mechanisms remain not yet fully understood, GLP-1R agonists actions in hepatic disease are likely multifactorial, involving CNS, adipose tissue, heart, and other tissues. Their efficacy in clinical trials positions them as valuable tools for managing metabolic liver diseases and, potentially, even complements to other hypothalamus-targeted interventions.

### Hepatic endocrine signals: *Fibroblast Growth Factor 21 (FGF21)*

FGF21 is a pleiotropic hormone primarily synthetized and secreted by the liver (Fig. 3), although its expression has also been reported in skeletal muscle, adipose tissue and pancreas [14, 163–165]. FGF21 exerts important endocrine functions by actively regulating glucose and lipid metabolism [63]. It most well-characterized action is the reducing of circulating glucose levels by enhancing its uptake into muscle and adipose tissue in an insulin-independent manner [166–168]. In this context, FGF21 acts as a potent insulin sensitizer during both prolonged fasting and the postprandial phase following overfeeding [168]. Circulating FGF21 levels are inversely correlated with BMI, hepatic fat accumulation, and fasting insulin levels, and are notably reduced in patients with T2D compared to healthy individuals [167]. One mechanism by which FGF21 improves hepatic glucose homeostasis is through stimulation of adiponectin production, which subsequently lowers hepatic ceramide concentrations [169].

Additionally, FGF21 exerts strong central actions, supported by the presence of its receptors in the hypothalamus (particularly in the VMH, PVN, ARC and SCN [170]) and hindbrain [14, 170–172]. It plays a vital role in metabolic regulation, particularly in response to stress and altered energy intake, influencing body weight, insulin sensitivity, and lipid levels [173–175]. In obese murine models, FGF21 has been shown to reduce body weight, blood glucose, and hepatic triglyceride content, at least partly through enhanced sympathetic outflow to BAT and WAT, thereby promoting thermogenesis and browning [176–179]. Consistent with these findings, genetic ablation of FGF21 aggravates obesity and impairs thermogenic responses, likely due to increased hypothalamic inflammation. This inflammatory profile has been associated with a shift in the expression of anti-thermogenic/thermogenic markers in the hypothalamus and elevated indicators of neuronal damage [180]. Further evidence from rat models shows that ICV infusion of FGF21 increases circulating levels of thyroid hormones, inducing WAT browning and contributing to body weight reduction [181].

Central FGF21 signaling mediates several systemic functions, including regulation of ketone bodies production, circadian rhythms, and fertility in female mice, which require the involvement of the transmembrane protein of β-klotho (a component of its receptor complex with the tyrosine kinase) [182, 183]. Loss-of-function studies of β-klotho have demonstrated that the central FGF21 actions, unlike its peripheral signaling in liver and adipose tissue, are crucial for modulating body weight, and circulating insulin and glucose levels in diet-induce obese mice [178]. Additional research shows that FGF21 influences corticotropin releasing factor (CRF) release within the CNS, thereby enhancing the sympathetic tone to peripheral tissues. This activation promotes WAT browning, BAT fatty acid oxidation and thermogenesis, and hepatic lipolysis and ketogenesis [177]. During prolonged fasting, fibroblast growth factor receptor 1 (FGFR1) plays a crucial role in maintaining glucose homeostasis [184]. Mechanistically, FGF21 activates MAPK/extracellular signal-related kinase (ERK) 1/2 pathway in an FGFR1-dependent manner, increasing CRF expression by activating the transcription factor cAMP response element-binding protein (CREB) in hypothalamic neurons [184]. These findings support the hypothesis that the therapeutic efficacy of FGF21-based pharmacological interventions for metabolic disorders may depend on their ability to activate FGFR1 and β-klotho in the CNS, particularly the hypothalamus (Table 1).

**Table 1:** Central hormonal signaling and its regulation of peripheral metabolic processes. Summary table highlighting the pancreatic and gastrointestinal hormones discussed in this Review and their principal metabolic effects on peripheral tissues relevant to systemic energy homeostasis

Secreted by	Hormones	Food intake	BAT	WAT		Liver	
			Thermogenesis	Browning	Lipolysis	Gluconeogenesis	Fatty acids oxidation
Pancreas	Insulin	↓	↑	↑	↓	↓	↓
	Glucagon	↓	↑	↑	-	↑	↑
	Amylin	↓	↑	↑	-	↓	↑
Intestine	GLP-1	↓	↑	↑	↑	↓	↑
Liver	FGF21	-	↑	↑	↑	↓	↑

Prolonged fasting or ketogenic diets induce hepatic FGF21 expression, significantly reducing body weight and enhancing insulin sensitivity in both murine models and primates. In the CNS, FGF21 stimulates sympathetic outflow via CRF, ultimately ameliorating whole-body metabolic function [177]. Through both direct and centrally mediated pathways, FGF21 promotes hepatic fatty acid oxidation and ketogenesis while suppressing hepatic lipid biosynthesis, thereby enhancing insulin sensitivity [185, 186]. In adipose tissue, FGF21 modulates adiponectin expression and secretion through PPARγ signaling, further potentiating insulin-sensitizing effects and stimulating WAT browning [186]. Notably, adiponectin-deficient mice are refractory to FGF21-induced changes in energy expenditure and insulin sensitivity, underscoring the conserved role of this pathways in mice [169]. Importantly, PPARα agonists elevate circulating FGF21 levels, and increased FGF21 concentrations have been observed in obese patients following 3 weeks of calorie restriction diet and in patients with rheumatoid arthritis after 7 days of fasting [187–189]. Interestingly, the weight-lowering effect of FGF21 treatment was blunted in mice with genetic deletion of the GDF15 receptor, while its glucose-lowering effects were preserved [190]. Thus, the beneficial effects of FGF21 on body weight and glucose homeostasis seem to be exerted through different mechanisms.

These findings highlight the therapeutic potential of FGF21-based interventions for metabolic dysfunctions. Several FGF21 analogues have been tested in clinical trials, showing promising results in patients with obesity, T2D, MASH, cirrhosis and fibrosis [191–196]. Benefits include significant reductions in fasting blood glucose, insulin, C-peptide and HOMA-IR levels, as well as improvements in hepatic steatosis and substantial reductions in hepatic lipid content when given alone [191–196]. Since GLP-1R are not expressed in the liver and GLP-1R agonists present no direct actions either in human hepatocytes or hepatic stellate cells [133, 197], their efficacy in treating metabolic liver diseases may involve indirect mechanisms, potentially mediated by FGF21 acting on different tissues. Indeed, liraglutide and exenatide stimulate hepatic FGF21 expression independently of feeding behavior in mice [198–200]. Similarly, liraglutide treatment increases FGF21 levels in patients with T2D [201]. Mechanistically, GLP-1R agonists may influence hepatic FGF21 expression through central pathways, including: (i) direct stimulation of hepatic autonomic projections; (ii) increased sympathetic tone to adipose tissue, triggering lipolysis and the release of free fatty acids that are potent activators of the hepatic PPARα, which in turn upregulates FGF21 expression; and (iii) activation of the hypothalamic-pituitary-adrenal axis, stimulating hepatic glucocorticoid receptors that, under certain circumstances, may activate FGF21 expression [197]. FGF21 modulation appears to mediate several biological effects attributed to GLP-1R agonists, including weight reduction, lipid homeostasis, and inhibition of hepatic gluconeogenesis and steatosis [200, 202]. These findings suggest that a combination therapy may help overcome obesity-induced FGF21 resistance. The GLP-1/FGF21 dual agonists have shown superior efficacy in promoting weight loss and glycemic control compared to monotherapy in diabetic mouse models [203]. Notably, in a small phase 2 cohort, patients with MASH treated with the combination of EFX, an FGF21 analogue, and GLP-1R agonists exhibited 65% reduction in liver fat, compared to only a 10% with GLP-1R agonists alone [204].

In summary, FGF21 agonists represent a promising therapeutic strategy for improving metabolic control in T2D and reversing liver fat accumulation, inflammation, and fibrosis in metabolic liver diseases. Despite these encouraging outcomes, most evidence derives from preclinical studies or early-phase clinical trial. Therefore, long-term efficacy and safety data in large human populations are eagerly awaited.

## Conclusion

Collectively, these findings highlight the hypothalamus as a central regulator of hepatic metabolism and a compelling therapeutic target in the context of metabolic and liver diseases. Disruptions in its neuroendocrine and autonomic pathways contribute substantially to metabolic dysfunctions, including MASLD and MASH. Accordingly, strategies that modulate hypothalamic signaling and its responsiveness to peripheral stimuli - such as insulin, glucagon, amylin, FGF21, and GLP-1 - represent promising avenues for restoring metabolic homeostasis and mitigate liver pathology. Advancing research on the gut-liver-brain axis and central-peripheral signaling networks will be essential for developing innovative, mechanism-based therapies that target both upstream regulators and downstream effectors of metabolic liver disorders. Furthermore, integrating these approaches with advanced machine-learning-guided peptide design holds considerable potential to optimize pharmacological profiles and achieve greater tissue specificity.

## Data Availability

No datasets were generated or analysed during the current study.

## Abbreviations

MASLD: Metabolic dysfunction-associated steatotic liver disease
NPY: Neuropeptide Y
GLP-1: Glucagon-like peptide-1
ARC: Arcuate nucleus of hypothalamus
VMH: Ventromedial nucleus of the hypothalamus
LHA: Lateral hypothalamic area
PVN: paraventricular nucleus of the hypothalamus
SF1: Steroidogenic factor 1
PEPCK: Phosphoenolpyruvate carboxykinase
PK: Pyruvate kinase
CRH: Corticotropin-releasing hormone
GABA: Gamma-aminobutyric acid
SCN: Suprachiasmatic nucleus
ERα: Estrogen receptor alpha
CNS: Central nervous system
NAFLD: Non-alcoholic fatty liver disease
MCH: Melanin-concentrating hormone
IR: Insulin receptor
BBB: Blood-brain barrier
ICV: Intracerebroventricular
AgRP: Agouti–related peptide
HFD: High fat diet
POMC: Proopiomelanocortin
BOLD: Blood oxygen level-dependent
T2D: Type 2 diabetes
CKD: Chronic kidney disease
PKA: Protein kinase A
AP: Area postrema
NTS: Nucleus of the solitary tract
CaMKKβ: Ca2+/calmodulin-dependent protein kinase kinaseβ
AMPK: AMP-activated protein kinase
BAT: Brown adipose tissue
FGF21: Fibroblast Growth Factor 21
GLP-1: Glucagon-like peptide 1
VLDL: Very low-density lipoprotein
MBH: Mediobasal hypothalamus
GLP-1R: GLP-1 receptor
NAc: Nucleus accumbens
Pdyn: Prodynorphin
VTA: Ventral tegmental area
WAT: White adipose tissue
NASH: Non-alcoholic steatohepatitis
CRF: Corticotropin releasing factor
FGFR1: Fibroblast growth factor receptor 1
ERK: Extracellular signal-related kinase
CREB: CAMP response element binding protein
